# Supplementary material for: Quantitative Transcriptome Analysis of Purified Equine Mast Cells Identifies a Dominant Mucosal Mast Cell Population with Possible Inflammatory Functions in Airways of Asthmatic Horses
Source: Int J Mol Sci. 2022 Nov 12;23(22):13976. doi: 10.3390/ijms232213976 (PMC9692376; doi:10.3390/ijms232213976)
Supplement: Supplementary file 1 [file ijms-23-13976-s001.zip › ijms-1999998-supplementary.pdf]

Supplementary Figures and Tables

Figure S1.

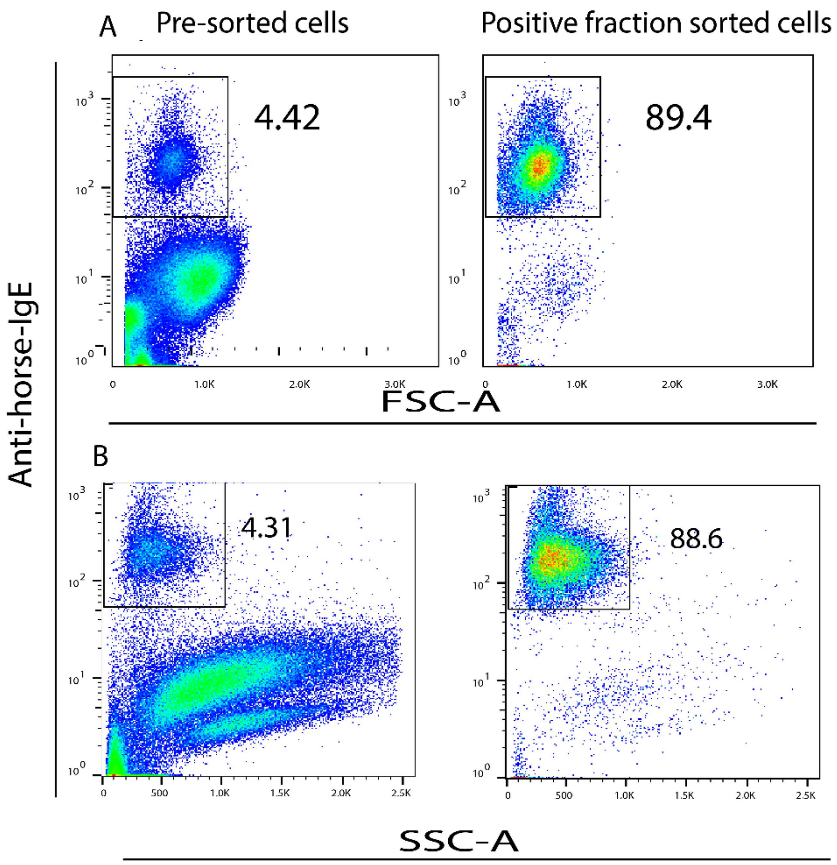

Figure S1. Presorted BALF cells and IgE-positive sorted BALF cells, including the forward scatter (FSC) and the side scatter (SSC). Analysis of anti-horse IgE-positive cells in BALF collected from an asthmatic horse using flow cytometry. In (A), IgE-positive cells (y-axis) with forward scatter (x-axis) and in (B), IgE-positive cells (y-axis) with side scatter (x-axis) are shown.

Figure S2.

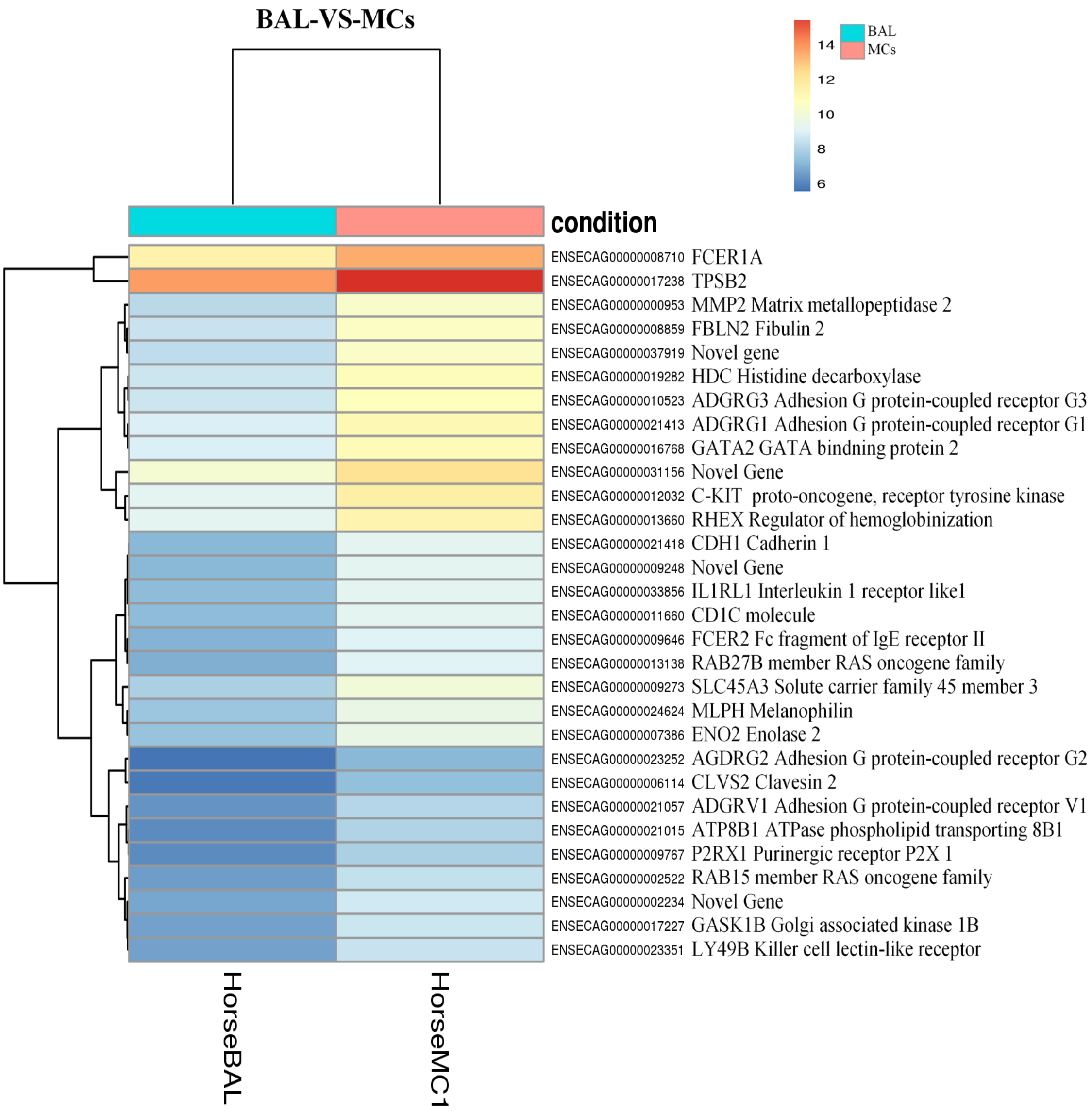

Figure S2. Bi-clustering heat map illustrating RNA-seq differential expression. These data come from the Dseq2 analysis of RNA-Seq data from total BALF cells (BAL) and isolated BALF MCs (MCs), which sorted the top 30 genes by their adjusted p-values. The colored legend indicates the condition (BAL or MCs) and the relative expression level.

Table S1.
Antibodies tested for cross reactivity with horse BALF MCs.[a]

Target molecule	Target species	Fluorochrome	Monoclonal antibody clone	Company
**CD117(c-kit)**	Human	PC7	104D2	eBioscience
**CD117(c-kit)**	Mouse	PC7	2B8	eBioscience
**CD117(c-kit)**	Mouse	APC	REA791	Miltenyi Biotec.
**FcεRI alpha**	Human	PE	AER-37 (CRA1)	eBioscience
**FcεRI alpha**	Mouse	PE	MAR-1	eBioscience

[a] List of monoclonal antibodies with low or no cross-reactive binding to horse BALF MCs, as determined by flow cytometry or MACS Quant Analyzer 10, using standard staining protocol.

Table S2. The most highly expressed genes from total horse BALF cells and from the three mast cell isolates MC1, MC2 and MC3. The top genes are ordered based on the top 100 genes in the MC1 isolate. The information was obtained from the common data analysis outlined in the Materials and Methods section.

	BALF cells (BALF sample 2)	MC1 (BALF sample 2)	MC2 (BALF sample 1)	MC3 (BALF sample 3)
Top expressed genes				
MT-CO1	29413.5831	41009.1666	27099.2988	95190.1416
MT-ATP6	11027.9631	20065.8006	12617.394	55115.6967
DRA	2344.3269	17906.9565	6758.9479	814.0243
B2M	18367.325	16219.7819	12477.8917	6740.4678
CD74	3531.8286	14764.8062	8191.8529	3494.6934
MT-ATP8	7202.0129	14661.4489	11414.0884	39015.4558
RPL37A	15444.3893	14616.6087	19607.7078	14350.7986
EEF1A1	4932.6501	9153.7189	10821.1493	8298.1784
TMSB4X	14808.5944	8951.0373	10425.9505	5240.0155
MT-CO2	11613.9032	8692.1977	5516.7553	22271.0356
FTH1	28251.8119	8277.1777	8023.8046	3655.4044
LGALS1	3867.4844	8205.7717	12901.8045	14845.4957
MT-ND1	3227.3398	7426.821	2843.3627	16742.5698
FTL	26772.8437	7208.7006	5512.6043	2124.792
RPS11	7231.2483	7173.8105	7403.1054	6567.1426
RPL13A	6702.7727	7066.2215	8088.914	6311.8633
MT-ND2	2540.6652	6246.2846	2795.6178	14609.7667
CST3	565.8169	6198.487	5139.6812	3753.9848
SRGN	4555.2375	5957.7922	7377.9738	5935.9011
RPS28	5480.1159	5501.0031	6117.2277	5380.4986
MT-ND3	2812.6265	5484.7986	3701.9221	8648.0827
RPS9	4777.3894	5255.437	5950.6155	5727.4605
RPS2	5253.7852	5056.2227	5048.0988	3712.9393
RPS29	4893.9921	5008.0098	7292.4365	4416.6278
RPL10	3755.7658	4977.8132	5997.076	5464.4795
MT-CYB	2298.6732	4713.5753	2031.3286	9808.3338
UBA52	3602.4615	4497.7648	3728.1857	3216.5139
APOE	22183.6157	4363.3079	3730.7079	1039.7364
MT-CO3	4074.6711	4312.2402	578.6597	9887.2979
RPS14	3983.8774	4263.1371	4630.9873	4564.1478
RPS16	4611.7064	4101.4146	4329.3582	4211.8415
VIM	4574.4093	4019.0991	5280.8344	4524.5363
ACTG1	4181.5759	4008.654	5041.1043	2687.4894
RPS23	2673.7217	4003.7441	5152.8207	5574.0417
RPL24	2882.4662	3952.8284	5398.3504	4987.7278
RPL35	3853.2129	3939.0645	4299.2906	4671.583
RPL37	3668.1705	3845.9311	4374.1342	3654.1183
RPLP0	3450.0812	3838.5416	4226.6212	4232.5212
IFI27	10106.4941	3629.8768	967.7666	792.4141
FAU	3488.2681	3479.0595	3845.0613	3587.8349

S100A4	1729.0353	3440.9262	6989.8292	7416.6548
RPS18	3262.8067	3410.7624	4062.7394	4066.3344
FCER1G	4710.4727	3387.2476	4240.6801	3591.4607
RPLP2	3327.3652	3370.5983	4166.4964	3546.4082
S100A11	2213.1348	3321.423	5787.0891	7670.9381
SYNGR2	1417.3198	3269.6095	2013.0855	1294.5899
RPS20	3168.4932	3248.8483	3396.3771	3636.7394
RPS26	2763.0711	3177.2914	3940.0881	3879.0775
ATP6V0C	5436.6992	3114.7646	2081.1349	1494.818
RPL31	2891.9624	3106.2985	3268.5299	3954.6219
IFI6	6808.9499	3035.3798	1261.044	541.9207
CYBA	6712.6237	3018.2843	2727.233	2618.1918
DQB	375.5303	2988.1509	418.3969	170.0539
CFL1	3523.2583	2975.4907	3444.1669	2629.8609
RPL7A	2243.6883	2950.3936	3473.3953	3638.8958
RPL13	1862.8981	2864.5135	3669.9886	3936.0674
RPS8	2789.2536	2861.5281	3535.18	3135.3605
LAPTM5	1790.5406	2824.9612	3710.8036	3114.6403
RPL39	3540.0233	2796.6998	2607.1654	2000.3933
CXCL16	1694.0473	2795.8787	998.0995	584.6483
RPS3	2226.4006	2782.9088	3936.3393	2225.5551
RACK1	1874.8985	2780.9184	3351.659	2826.4767
IFI30	790.0586	2753.2704	1491.6262	743.0892
PSAP	7299.2739	2731.4587	1889.8737	518.1595
RPS21	2983.3361	2724.9936	3862.9793	2353.7604
PFN1	3345.8616	2688.7848	2787.8975	2728.9845
RPL18A	2876.9272	2667.6188	2440.3474	1850.2689
RPL7	1667.6187	2652.5088	4421.0739	3791.1485
TYROBP	4206.2112	2647.7416	1977.8724	1383.3962
RPS15A	2724.0433	2641.5977	2680.9502	2460.9648
RPS6	2064.7755	2511.6073	3187.4761	3360.1335
RPL10A	2201.2484	2473.5673	2875.8551	2925.8774
RPL26	2102.6235	2462.0736	3145.9747	3044.8894
BANF1	2570.2588	2433.6456	2485.7911	1967.6654
LTC4S	109.4815	2413.6191	4507.9367	4301.0991
RPS3A	1301.0475	2398.3456	2920.6022	2279.0671
RPL27A	2106.2484	2396.9132	3247.9305	2316.4724
RPL18	2588.0829	2386.9458	2512.42	2152.2005
ACTB	3374.6002	2386.1525	3427.2546	2265.7028
GABARAP	1782.6863	2364.9933	2553.4443	1894.6148
UBB	2183.8752	2357.6283	2621.4363	1917.7659
H3-3B	1386.157	2339.7332	2765.2267	1668.6622
RPS13	1973.9772	2317.5161	3123.1092	2124.4465
RPSA	2984.189	2252.3091	2075.9447	1595.0746
RPL38	2241.3072	2229.8482	2467.3981	1769.8209
RPL9	1831.2817	2229.7961	3012.0091	1823.0029
RNASE6	3962.4337	2193.4279	835.5649	166.4551
MYL6	3237.489	2189.4647	3120.9925	2976.3022

ALOX5AP	1887.7095	2174.9508	2890.2818	2307.23
RPL27	2175.7931	2167.9698	2732.5585	2797.2952
RPS25	1859.6021	2153.0814	2829.9172	2580.5886
LY6E	3035.4682	2143.0504	2284.1401	1428.5346
RPS7	1839.4933	2102.3407	2652.7107	2112.2468
RPL14	1605.9125	2083.8305	3123.6833	2753.2739
RPL3	1518.4701	2051.3819	2180.7268	1960.9311
RPL19	1867.1773	2036.1283	2634.2234	2777.0474
TPT1	2328.544	2006.6031	2379.8926	1551.6285
DRB	378.8709	1990.7242	685.249	198.2401
RPL8	1744.6062	1919.9396	2167.1875	1944.1248
RPL28	2325.6156	1855.9381	2057.0447	2182.6842

Table S3.
Transcript levels for additional important MC-expressed genes. The number of normalized counts (TPM) was given for each gene (obtained from GENEWIZ).

Gene	BALF MCs	Total BALF cells	Functions
FBLN2	45	1	Tissue development and remodeling (Extra cellular matrix glycol protein)
ECE1	17	1	Proteolytic processing of endothelin-1,2 and 3
ENO2	101	1.1	Glucose metabolism
ANXA3	49	1.7	Regulation of cellular growth and in signal transduction pathways
ADGRG3	178	3	Involved in G protein-coupled receptor signaling pathway and regulation of cell migration
MT3	49	3	It plays an important role in zinc and copper homeostasis, and is induced under hypoxic conditions,
IGFBP7	139	4	Regulate the IGF availability in body fluids and tissues
CASP6	61	5	Apoptosis
RGS13	136	6	Regulator of G-protein signaling 13
PLPP1	62	6	Synthesis of glycolipids and in phospholipase D-mediated signal transduction,
ZFR2	58	9	Zinc finger RNA bindning protein 2
LAPTM4B	117	9	Regulation of lysosomal membrane permeability; and regulation of lysosome organization
RHEX	523	13	Acts as a signaling transduction factor of the EPO-EPOR signaling pathway promoting erythroid cell differentiation
MAP4K1	73	18	Involved in several processes, including JNK cascade; cellular response to phorbol 13-acetate 12-myristate; and protein phosphorylation.
HMGCR	176	27	The rate-limiting enzyme for cholesterol synthesis
ICMA2	164	28	This protein may play a role in lymphocyte recirculation by blocking LFA-1-dependent cell adhesion
LYL1	71	28	Hematopoiesis
SQLE	134	29	Catalyzes the first oxygenation step in sterol biosynthesis
ITGAE	185	30	The chief ligand for αEβ7 is E-cadherin, a cellular adhesion molecule (CAM) found on epithelial cells
FDPS	168	46	Catalyzes the production of geranyl pyrophosphate and farnesyl pyrophosphate from isopentenyl
SERINC3	153	48	Predicted to enable L-serine transmembrane transporter activity,
LAT2	166	48	Involved in FCER1(high affinity immunoglobulin epsilon receptor)-mediated signaling in mast cells

ITGB7	155	53	Interact with ITGAE and EED
LAT	630	89	Signaling, T-cell, NK cell and mast cell development

Table S4.
Transcript levels for the S100 family genes. The number of normalized counts (TPM) was given for each gene (obtained from GENEWIZ).

	**Gene**	**BALF MCs**	**Total BALF cells**
**S100 family genes[a]**	S100A4	3440	1729
	S100A11	3321	2213
	S100A10	1130	1157
	S100P	1625	8969
	S100A6	753	506

[a]A family of calcium-binding cytosolic proteins.

Table S5.
Transcript levels for the FKBP family of genes. The number of normalized counts (TPM) was given for each gene (obtained from GENEWIZ).

	**Gene**	**BALF MCs**	**Total BALF cells**
**FKBP Family genes[a]**	FKBP8	224	238
	FKBP2	143	159
	FKBP4	67	46
	FKBP3	44	36
	FKBP5	44	27

[a]Immunophilins consist of a family of highly conserved proteins binding with immunosuppressive drugs.

Table S6.
Transcript levels for CD molecules. The number of normalized counts (TPM) was given for each gene (obtained from GENEWIZ).

	**Gene**	**BALF MCs**	**Total BALF cells**
**CD molecules[a]**	CD4	156	111
	CD9	597	131
	CD22	15	1
	CD37	781	706
	CD53	766	984
	CD81	302	124
	CD83	446	50
	CD96	38	18
	CD99	636	516

[a] Cell surface proteins
